# Keratinocyte-Expressed Podoplanin is Dispensable for Multi-Step Skin Carcinogenesis

**DOI:** 10.3390/cells9061542

**Published:** 2020-06-24

**Authors:** Marko Sesartić, Kristian Ikenberg, Sun-Young Yoon, Michael Detmar

**Affiliations:** 1 Institute of Pharmaceutical Sciences, Swiss Federal Institute of Technology, ETH Zurich, Vladimir-Prelog-Weg 3, HCI H303, 8093 Zürich, Switzerland; marko.sesartic@pharma.ethz.ch (M.S.); youcan26@skku.edu (S.-Y.Y.); 2 Department of Pathology and Molecular Pathology, University and University Hospital Zurich, 8091 Zurich, Switzerland; kristian.ikenberg@usz.ch; 3 School of Pharmacy, Sungkyunkwan University, Suwon-si, Gyeonggi-do, Korea

**Keywords:** carcinogenesis, podoplanin, lymphangiogenesis, keratinocytes

## Abstract

Podoplanin is a small transmembrane mucin-like glycoprotein that plays a crucial role in the development of the lung, heart and lymphatic vascular system. Its expression is upregulated in several types of human carcinomas and podoplanin levels in squamous cell carcinomas (SCCs) of the oral cavity and the lung correlate with cancer invasiveness, lymph node metastasis and shorter survival time of patients, indicating that podoplanin promotes tumor progression. However, its role during the early stages of carcinogenesis remain unclear. We generated mice with a specific deletion of podoplanin in epidermal keratinocytes (K5-Cre;Pdpn^flox/flox^ mice) and subjected them to a multistep chemical skin carcinogenesis regimen. The rate of tumor initiation; the number, size and differentiation of tumors; and the malignant transformation rate were comparable in K5-Cre;Pdpn^flox/flox^ mice and Pdpn^flox/flox^ control mice. However, tumor cell invasion was reduced in K5-Cre;Pdpn^flox/flox^ mice, in particular single cell invasion. Quantitative immunofluorescence analyses revealed that peritumoral lymphangiogenesis was reduced in K5-Cre;Pdpn^flox/flox^ mice, whereas there were no major changes of tumor-associated immune cell subpopulations. Thus, keratinocyte-expressed podoplanin is dispensable for the early steps of skin carcinogenesis but contributes to the progression of established tumors.

## 1. Introduction

Podoplanin (Pdpn) is a small type I transmembrane mucin-like glycoprotein known under several different names, including PA2.26, gp38, T1α, D2-40, and aggrus [1]. It is expressed in a number of adult tissues, including glomerular podocytes (hence its name), type I alveolar cells, mesothelial cells, choroid plexus cells, glia cells, sebaceous glands, different types of fibroblasts, and lymphatic endothelial cells (LECs) [2,3]. In normal skin, podoplanin is expressed by the basal cell layer of sebaceous glands and the outer root sheath keratinocytes of hair follicles, but not by the interfollicular epidermis [4,5]. Podoplanin is crucial for the normal embryonic development and function of the lungs, the heart and the lymphatic vascular system, as demonstrated by studies in podoplanin knockout mice [6,7,8].

The upregulation of podoplanin expression has been reported in both human cancers and in experimentally generated animal tumors. Podoplanin expression is upregulated in a number of different human cancers, including germ cell tumors, tumors of the central nervous system and squamous cell carcinomas (SCCs) of the oral cavity, skin and the lung [9,10,11,12,13]. Cancer cell expression of podoplanin in SCCs of the oral cavity and the lung correlates with increased cancer invasiveness, lymph node metastasis and the shorter survival time of patients [9,11,14]. Interestingly, podoplanin expression is often limited to the outer edge of tumors, suggesting that the stromal cells might be able to induce podoplanin expression in the transformed epithelial cells through secretion of cytokines and growth factors [3,12,15,16]. In a breast carcinoma xenograft animal model, podoplanin overexpression promoted tumor-associated lymphangiogenesis, invasiveness and lymph node metastasis [9]. Additionally, in the mouse model of multistep chemically induced skin carcinogenesis, podoplanin expression was upregulated by both the transformed keratinocytes and the stromal cells [17]. In the human SCC A431 cell line, podoplanin-expressing cells form colonies with high efficiency and have high tumorigenicity in nude mice [18]. Podoplanin was also reported to be involved in the regulation of cytoskeletal organization and cell motility [19,20]. Together with the localization of podoplanin at the invasive front of SCC tumors [21], podoplanin appears to play an important role in the detachment and invasion of tumor cells into the adjacent tumor stroma, which are crucial steps towards metastasis.

While there is evidence that increased podoplanin expression might play an important role in tumor progression and metastasis, it is unclear whether podoplanin is necessary for tumor progression and the relevance of cancer cell expressed podoplanin for the early stages of carcinogenesis remains unclear. The two-stage chemically induced carcinogenesis model is one of the best characterized in vivo models for the study of the sequential development of tumors [22]. In this model, carcinogenesis occurs as a consequence of a single dose of the carcinogen, 7,12-dimethylbenz (α) anthracene (DMBA), and sequential treatments with the promoting agent, 12-*O*-tetradecanoyl-phorbol-13-acetate (TPA). The cancers develop through a distinctive three-step process, namely initiation, promotion and progression, mimicking the human cancer formation from the initiating stage onwards [23]. In the present study, we investigated the role of epidermal cell expressed podoplanin in tumor initiation and growth in the two-step mouse chemical skin carcinogenesis model, using epidermis-specific podoplanin knockout (K5-Cre;Pdpn^flox/flox^) mice. We found that keratinocyte-expressed podoplanin is dispensable for tumor initiation and multiplicity in a mouse chemical skin carcinogenesis model. However, tumor invasion and peritumoral lymphangiogenesis were reduced in K5-Cre;Pdpn^flox/flox^ mice.

## 2. Materials and Methods

### 2.1. Ethics Statement

All animals were housed and fed according to federal guidelines, and animal experimental procedures were conducted according to animal protocol (ZH198/15) approved by the local veterinary authorities (Kantonales Veterinäramt Zürich, Zurich, Switzerland).

### 2.2. Mouse Model

The generation of K5-Cre;Pdpn^flox/flox^ mice was previously described [5]. Briefly, we crossed mice constitutively expressing keratin 5 promoter-driven Cre recombinase (K5-Cre), obtained from the MMRRC Repository (University of Missouri, MO, USA), with Pdpn exon 2 floxed (Pdpn^flox/flox^) mice to generate epidermis-specific podoplanin K5-Cre;Pdpn^flox/flox^ knockout mice and Pdpn^flox/flox^ control mice on the C57BL/6 background. The mice were bred as male K5-Cre;Pdpn^flox/flox^ mice with female Pdpn^flox/flox^ mice, producing Pdpn^flox/flox^ control mice and K5-Cre;Pdpn^flox/flox^ epidermis-specific podoplanin knockout mice. As reported previously, these mice show enhanced hair follicle growth after depilation-induced hair follicle regeneration, but otherwise have no major changes of epidermal differentiation and architecture [5].

### 2.3. Chemical Carcinogenesis

An established two-step chemical skin carcinogenesis protocol was used to induce tumors [22]. Female Pdpn^flox/flox^ control and K5-Cre;Pdpn^flox/flox^ knockout mice were shaved on their back skin under isoflurane anesthesia at 8 weeks of age. Two days later, mutations in the DNA were induced by topical application of 25 µg of the carcinogen 7,12-dimethylbenz (α) anthracene (DMBA), dissolved in 200 µL of acetone, on the back skin of mice anesthetized with ketamine-medetomidine. After 7 days, 20 weekly topical applications of 7.5 µg of 12-*O*-tetradecanoyl-phorbol-13-acetate (TPA), dissolved in 200 µL of acetone were performed on the back skin under isoflurane anesthesia. The back skin was shaven periodically to ensure topical application of TPA onto the skin. Tumor growth was monitored once per week in a blinded manner. Raised lesions of a minimum diameter of 1 mm present for at least 1 week were recorded as tumors. Morphologically heterogeneous and infiltrative tumors over 5 mm in diameter were scored as SCCs and mice were sacrificed at the latest 6 weeks after the SCC diagnosis. After sacrifice, the SCC diagnosis was confirmed by histological analysis in each case. Mice that did not develop SCCs were sacrificed after a maximum of 52 weeks after DMBA treatment. Mice were sacrificed with an overdose of anesthesia (160 mg/kg ketamine; 0.4 mg/kg medetomidine), and the tissue samples were fixed in 4% paraformaldehyde overnight and embedded in paraffin.

### 2.4. Hematoxylin-Eosin Staining

Paraffin sections of the control and K5-Cre;Pdpn^flox/flox^ tumors were de-paraffinized, rehydrated and incubated with hematoxylin solution Gill III (Merck, Darmstadt, Germany), washed with water and 0.1% hydrochloric acid and stained with 0.5% aqueous eosin Y (Merck). After ethanol and xylene dehydration, the sections were mounted using Eukitt mounting medium (Sigma-Aldrich, St. Louis, MO, USA).

### 2.5. Immunofluorescence Staining

Paraffin sections of control and K5-Cre;Pdpn^flox/flox^ tumors were de-paraffinized, rehydrated and subjected to antigen-retrieval using sodium citrate buffer. The sections were blocked with a buffer consisting of 5% donkey serum, 0.2% bovine serum albumin and 0.3% Triton X-100 in PBS for 1 h at RT. Next, sections were incubated with primary antibodies overnight at 4 °C and, after several washes in 0.5% PBST, incubated with secondary antibodies for 30 min at RT (room temperature). Primary antibodies were as follows: rabbit anti-mouse LYVE1 (Angiobio, San Diego, CA, USA), goat anti-mouse podoplanin (R&D Systems, Minneapolis, MN, USA), rabbit anti-mouse cytokeratin 5 (Abcam, Cambridge, United Kingdom), rat anti-mouse MECA-32 (BD Biosciences, Franklin Lakes, NJ, USA), polyclonal goat anti-mouse CD45 (R&D Systems), and rat anti-mouse CD68 (eBioscience, San Diego, CA, USA). Alexa Fluor 488- and Alexa Fluor 594-conjugated secondary antibodies and Hoechst 33342 for nuclear staining were purchased from Invitrogen (Carlsbad, CA, USA). Slides were mounted with Mowiol mounting medium.

**Morphometric analyses**. Tissue sections were imaged on an Axioskop2 mot plus microscope (Carl Zeiss, Oberkochen, Germany) with an AxioCam MRc camera (Carl Zeiss) and a Plan-APOCHROMAT ×10 or ×20 objective (Carl Zeiss) using AxioVision software 4.8 (Carl Zeiss), or with a Panoramic 250 Flash III (3D Histech, Budapest, Hungary) slide scanner using CaseViewer 2.3 software (3D Histech). High-magnification images were obtained using a Zeiss LSM 710 FCS confocal microscope. Hematoxylin-eosin (H&E)-stained tumor sections were evaluated by a pathologist in a semi-quantitative way and scored categorically for invasion, differentiation, the number of dysplastic cells, necrosis and inflammatory infiltration in a blinded fashion. For the immunofluorescent stainings of peri-tumoral lymphatic vessels, the area of interest was defined as the area stretching from the tumor border to 500 µm away from it and the LYVE1 positive area (in % of total area of interest) and LYVE1-positive lymphatic vessels were counted using ImageJ (NIH, Bethesda, USA) software version 1.49 v in a blinded fashion.

### 2.6. Isolation of Primary Keratinocytes

The method for the isolation of primary mouse keratinocytes has been previously described [24]. Briefly, whole skin from control and K5-Cre;Pdpn^flox/flox^ mice was collected at postnatal day 2–4 and digested in 0.8% trypsin solution (Sigma-Aldrich) with the dermis facing down with rotary shaking at 75 rpm for 1 h at 37 °C. The epidermis was peeled away, minced using scissors and incubated in Dulbecco’s Modified Eagle Medium supplemented with 30 µg/mL DNase solution (AppliChem, Darmstadt, Germany) and antibiotic/antimycotic solution for 30 min at 37 °C under constant rotation. Afterwards, cells were passed through 100 µm strainers and washed in PBS supplemented with 1% FBS and 2 mM ethylenediaminetetraacetic acid (EDTA). Subsequently, the cells were spun down (7 min, 400 g) and seeded on 25 µg/mL type IV collagen (collagen from human placenta, Sigma-Aldrich)-coated 6-well plates. Cells were cultured in complete keratinocyte culture media consisting of supplemented defined keratinocyte-SFM (Invitrogen) and supplemented Minimum Essential Medium Eagle (MEM; Sigma-Aldrich) at a ratio of 2:1 with 10 ng/mL epidermal growth factor (Sigma-Aldrich). The defined keratinocyte-SFM (Invitrogen) was supplemented with 0.2% growth supplement (Invitrogen), 1% penicillin/streptomycin (Invitrogen), and 10 − 10 M cholera toxin (Sigma-Aldrich). KGF (keratinocyte growth factor)-medium (MEM, Sigma-Aldrich) was supplemented with 5 µg/mL insulin, 10 µg/mL transferrin, 1.4 µg/mL phosphoethanolamine, 10 mM ethanolamine (all from Sigma-Aldrich), 0.36 µg/mL hydrocortisone (Calbiochem, Darmstadt, Germany), 1% glutamine (Invitrogen), 1% penicillin/streptomycin (Invitrogen), 8% chelated FCS (Bio-Rad, Hercules, CA, USA), and 6.6 µg/mL CaCl2 (Merck). After 3 days in culture, the cultures consisted of more than 90% primary keratinocytes, defined as CD45-, CD90- and integrin α6^+^ cells, confirmed by flow cytometry FACS (CytoFLEX, Beckman Coulter, Brea, CA, USA).

### 2.7. Proliferation Assays

Proliferation assays were performed as described previously [25]. Briefly, 2500 primary keratinocytes from Pdpn^flox/flox^ control and K5-Cre;Pdpn^flox/flox^ mice were seeded into 96-well plates in complete keratinocyte culture medium (Lonza, Basel, Switzerland) coated with 25 µg/mL type IV collagen (collagen from human placenta, Sigma-Aldrich) and were incubated overnight in serum-reduced medium containing 1% FBS and then returned to full medium. After incubation for 72 h, 100 µg/mL 4-methylumbelliferyl heptanoate (MUH, Sigma-Aldrich) was added and the fluorescence intensity, corresponding to the number of viable cells (Stadler et al., 1989), was measured on a SpectraMax reader (Molecular Devices, San Jose, CA, USA) at 355 nm excitation and 460 nm emission. For each condition, at least quintuplicates were analyzed and the assay was performed 3 times.

### 2.8. Scratch Wound Healing Assay

Migration assays were performed as described previously [26]. Shortly, primary keratinocytes from Pdpn^flox/flox^ control and K5-Cre;Pdpn^flox/flox^ mice were cultured to confluence on 25 µg/mL type IV collagen-coated 24-well plates. Cells were incubated overnight in serum-reduced medium containing 1% FBS. Cell layers were scratched with a 200 µL sterile pipette tip and were then incubated in complete media. To monitor scratch closure, images were taken using a Zeiss AxioVert 200M microscope equipped with a 5× magnification lens and analyzed using the TScratch software version 1.0 [26].

### 2.9. Statistical Analysis

All statistical analyses were performed using the GraphPad Prism 8 software (GraphPad Software Inc., San Diego, CA, USA). For the comparison of continuous variables between two groups, a two-tailed unpaired Student’s *t*-test was applied. For repeated measurements, a two-way ANOVA with Bonferroni post-test was applied, and for comparison of categorical data the Fisher’s exact test was used. A *p* value of *p* < 0.05 was considered significant (*p* < 0.05 *, *p* ≤ 0.01 **, *p* ≤ 0.001 ***).

## 3. Results

### 3.1. Keratinocyte-Expressed Podoplanin Is Dispensable in the Early Stages of Skin Carcinogenesis

**Figure 1:**
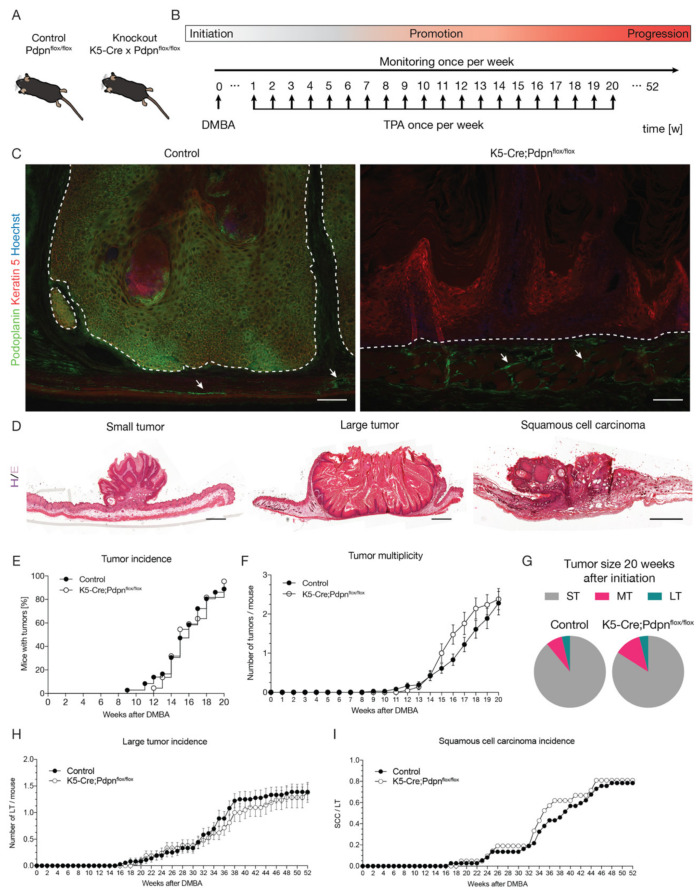
Tumor-expressed podoplanin is dispensable for tumor initiation, growth and malignant transformation in the two-stage chemical carcinogenesis model. (**A**) Epidermis-specific podoplanin knockout out mice were made by crossing control Pdpn^flox/flox^ and epidermis-specific podoplanin knockout K5-Cre;Pdpn^flox/flox^ mice. (**B**) Carcinogenesis was initiated with a single topical application of the tumor initiator DMBA (25 µg), followed by 20 weekly topical applications of the tumor promoter TPA (7.5 µg). Mice were monitored once per week and sacrificed 6 weeks after squamous cell carcinomas (SCC) diagnosis or at the latest 52 weeks after initiation. (**C**) Immunofluorescence images of tumors from control and K5-Cre;Pdpn^flox/flox^ mice stained for podoplanin (green) and keratin 5 (red). Nuclei are stained by Hoechst (blue). Dashed white line separates the tumor cells (above) and the tumor stroma (below). White arrows indicate lymphatic vessels. Scale bar: 200 µm. (**D**) Micrographs of H&E stainings of representative small tumor, large tumor and squamous cell carcinoma morphology. Scale bar: 1 mm. (**E**) Quantification of tumor incidence in control and K5-Cre;Pdpn^flox/flox^ mice as percent of mice bearing at least one tumor with a minimum diameter of 1 mm present for more than one week (*n* = 22–35 animals per group, Kaplan-Meier). (**F**) Quantification of tumor multiplicity in control and K5-Cre;Pdpn^flox/flox^ mice as the average number of tumors per mouse (*n* = 22–35 animals per group, two-way ANOVA). (**G**) Size distribution of small (ST, between 1 and 3 mm diameter), medium (MT, between 3 and 5 mm diameter) and large tumors (LT, over 5 mm in diameter) in control and K5-Cre;Pdpn^flox/flox^ mice 20 weeks after DMBA treatment. (**H**) Quantification of large tumors (>5 mm in diameter) incidence in control and K5-Cre;Pdpn^flox/flox^ mice (*n* = 22–35 animals per group, two-way ANOVA). (**I**) Quantification of SCC incidence in control and K5-Cre;Pdpn^flox/flox^ mice expressed as the product of SSC and large tumors (over 5 mm in diameter) numbers (*n* = 22–35 animals per group, two-way ANOVA).

In normal skin, podoplanin is expressed by the lymphatic vessels, the basal cell layer of sebaceous glands and the outer root sheath of anagen hair follicles, but not by the interfollicular epidermis [5,17]. Mouse basal keratinocytes and dermal fibroblasts upregulate podoplanin expression under proliferative conditions, such as wound healing, psoriasis and phorbol ester 12-*O*-tetradecanoylphorbol 13-acetate (TPA)-induced inflammation [4,27,28]. To investigate the biological role of podoplanin in the early stage of carcinogenesis, we generated epidermis-specific podoplanin knockout mice, K5-Cre;Pdpn^flox/flox^, as described previously [5] (Figure 1A). Control Pdpn^flox/flox^ and K5-Cre;Pdpn^flox/flox^ epidermis-specific podoplanin knockout mice were subjected to the two-step chemical carcinogenesis protocol. The protocol consisted of a single topical application of the carcinogen 7,12-dimethylbenz (α) anthracene (DMBA) to the back skin, followed by 20 weekly topical applications of the tumor promoter TPA (Figure 1B).

Immunofluorescence stainings revealed strong podoplanin expression in both the tumors and SCCs of the control mice, while there was no detectable podoplanin staining in any of the tumors of K5-Cre;Pdpn^flox/flox^ mice (Figure 1C). However, the lymphatic vessel staining for podoplanin was maintained in the K5-Cre;Pdpn^flox/flox^ mice (Figure 1C). These findings confirmed the efficiency and specificity of the podoplanin deletion in epidermal keratinocytes. Tumor growth was monitored weekly and raised lesions over 1 mm in diameter persisting for more than 1 week were counted. Surprisingly, tumor incidence, that is the proportion of mice bearing at least one tumor, did not significantly differ between the control and K5-Cre;Pdpn^flox/flox^ mice. Initially, the K5-Cre;Pdpn^flox/flox^ mice showed a 3-week delay in the formation of the first tumors compared to the control mice, with the first tumors appearing 12 weeks after the DMBA treatment in K5-Cre;Pdpn^flox/flox^ mice compared to 9 weeks in the control mice. After the last application of the tumor promoter TPA, 95% of K5-Cre;Pdpn^flox/flox^ mice had tumors, as compared to 89% of control mice (Figure 1E). Overall, there was no significant difference in the kinetics of tumor incidence between the control and K5-Cre;Pdpn^flox/flox^ mice. Similarly, tumor multiplicity, defined as the average number of tumors per mouse, did not show any significant difference between the control and K5-Cre;Pdpn^flox/flox^ mice. The K5-Cre;Pdpn^flox/flox^ mice had a slightly higher average number of tumors per mouse compared to the control mice from week 15 to week 19 of TPA application. However, after the last TPA application, there was no difference in tumor multiplicity (Figure 1F). Tumors were scored according to their size as either small (between 1 and 3 mm diameter), medium (between 3 and 5 mm diameter) or large tumors (over 5 mm in diameter) (Figure 1D). There was no difference in the size distribution of tumors between the control and K5-Cre;Pdpn^flox/flox^ mice after the last tumor promoter application at 20 weeks (Figure 1G). In line with these results, there was no significant difference in the incidence of large tumors (>5 mm) between the control and K5-Cre;Pdpn^flox/flox^ mice (Figure 1H). We scored morphologically heterogeneous and infiltrative tumors over 5 mm in diameter as SCCs and sacrificed the mice 6 weeks after their first detection. The control mice developed the first SCC 17 weeks after DMBA initiation compared to 19 weeks in K5-Cre;Pdpn^flox/flox^ mice (Figure 1I). However, the overall incidence of SCCs did not significantly differ between the control and K5-Cre;Pdpn^flox/flox^ mice (Figure 1I). Taken together, these data suggest that keratinocyte-expressed podoplanin is dispensable for skin tumor initiation, growth and malignant transformation in the two-stage chemical carcinogenesis model.

### 3.2. Tumor Cell Expressed Podoplanin Promotes Tumor Cell Invasion

**Figure 2:**
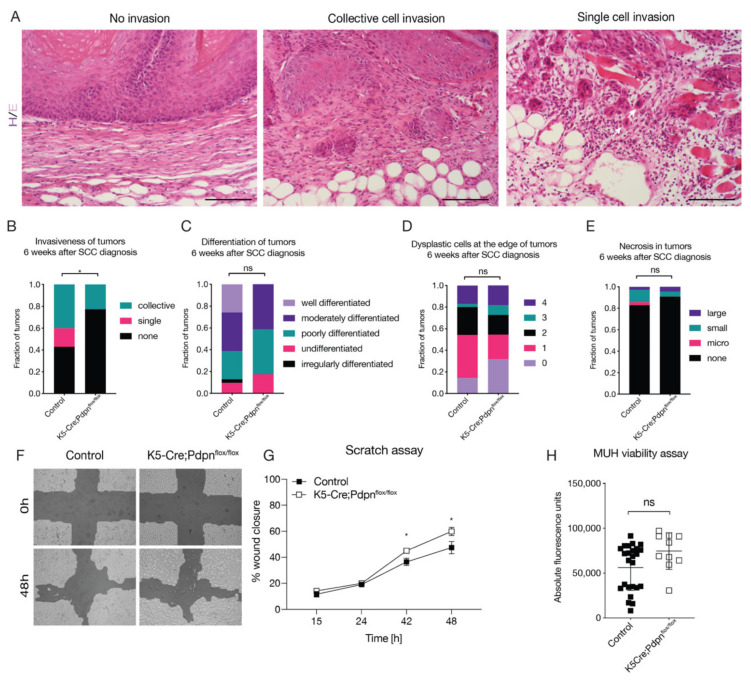
Absence of cancer cell expressed podoplanin reduces cell invasion. (**A**) Representative micrographs of non-invasive tumor, collective cell invasion and single cell invasion through the basement membrane. Scale bar: 200 µm. Semi-quantitative evaluation of (**B**) tumor invasiveness, (**C**) tumor differentiation, (**D**) dysplastic cells and (**E**) tumor necrosis in tumors of control and K5-Cre;Pdpn^flox/flox^ mice 6 weeks after SCC diagnosis (*n* = 22–35 tumors per group, Fisher’s exact test). (**F**) Representative pictures of scratch migration of control and K5-Cre;Pdpn^flox/flox^ keratinocytes. Quantification of cell migration (**G**) and cell proliferation (**H**) of primary keratinocytes isolated from control and K5-Cre;Pdpn^flox/flox^ mice (*n* = 8 wells per group for migration, 10–25 wells per group for proliferation, one of three similar experiments shown). Data are presented as mean ± SD. * *p* < 0.05.

We and others have previously reported that cancer cell overexpression of podoplanin promotes cancer cell invasiveness [9,19]. Cancer cell expressed podoplanin has been reported to promote both single cell invasion and epithelial-mesenchymal transition (EMT), as well as collective cell invasion [19,20,29,30,31]. We investigated tumor invasiveness by scoring histopathological sections of tumors as either having collective cell invasion, single cell invasion or no invasion with respect to the basement membrane (Figure 2A). We found that K5-Cre;Pdpn^flox/flox^ mice had significantly less invasion compared to control mice (Figure 2B). Interestingly, only control mice displayed single cell invasion. Next, we evaluated tumor differentiation as an indicator of tumor aggressiveness. Tumors were scored as either well differentiated, moderately differentiated, poorly differentiated, undifferentiated or irregularly differentiated. No significant difference between the control and K5-Cre;Pdpn^flox/flox^ mice was observed (Figure 2C). Similarly, the number of dysplastic cells, scored on a scale of 0 to 4, from least to most, was not significantly different between the control and K5-Cre;Pdpn^flox/flox^ mice (Figure 2D). Overall, very little necrosis was observed in both the control and K5-Cre;Pdpn^flox/flox^ mice (Figure 2E). We also isolated primary epidermal keratinocytes from the control and K5-Cre;Pdpn^flox/flox^ mice and performed in vitro migration and proliferation assays. Isolated primary keratinocytes from the control and K5-Cre;Pdpn^flox/flox^ mice were cultured for four days until confluence. In the scratch migration assay, K5-Cre;Pdpn^flox/flox^ keratinocytes migrated significantly faster than the control keratinocytes (Figure 2F,G). Even though this result was unexpected, the two-dimensional scratch assay likely does not reflect the complex tumor microenvironment where transformed cells need to invade through a complex three-dimensional extracellular matrix that has been remodeled by the tumor stroma. There was no significant difference in the proliferation between the control and knockout keratinocytes (Figure 2H).

### 3.3. Tumor Cell Expressed Podoplanin Promotes Peritumoral Lymphangiogenesis

**Figure 3:**
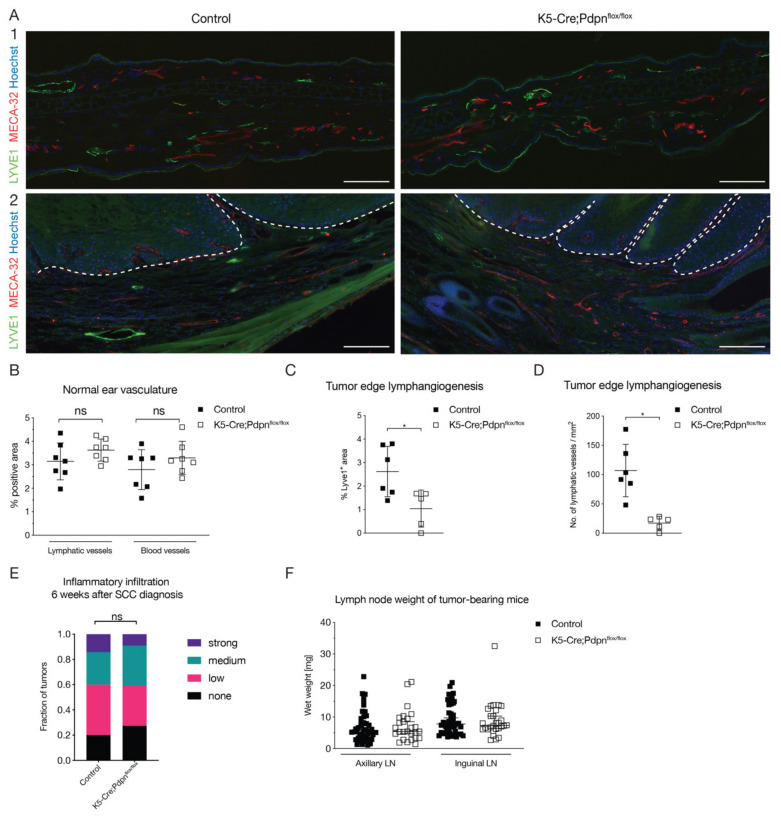
Tumor cell expressed podoplanin promotes peritumoral lymphangiogenesis. (**A**) Immunofluorescence images of (**1**) normal ears and (**2**) SCCs from control and K5-Cre;Pdpn^flox/flox^ mice stained for LYVE1 (green) and MECA-32 (red). Nuclei are stained by Hoechst (blue). Dashed line separates the tumor cells (above) and the tumor stroma (below). Scale bar: 100 µm. (**B**) Quantification of lymphatic (LYVE1^+^) and blood vessel (MECA-32^+^) area in normal ears of control and K5-Cre;Pdpn^flox/flox^ mice (*n* = 7 ears per group, Mann–Whitney test). Quantification of the LYVE1^+^ area as % of total area (**C**) and number of LYVE1^+^ lymphatic vessels per mm^2^ (**D**) in tumors of control and K5-Cre;Pdpn^flox/flox^ mice, measured in the peritumoral area stretching from the tumor edge to 500 µm away from it (*n* = 4–6 tumors per group, Mann–Whitney test). (**E**) Semi-quantitative assessment of inflammatory infiltration in H&E-stained sections of tumors of control and K5-Cre;Pdpn^flox/flox^ mice 6 weeks after SCC diagnosis (*n* = 22–35 tumors per group, Fisher’s exact test). (**F**) Wet lymph node weights of tumor-bearing control and K5-Cre;Pdpn^flox/flox^ mice 6 weeks after SCC diagnosis (*n* = 22–35 lymph nodes per group, Mann–Whitney test). Data represent mean ± SD. * *p* < 0.05.

Cancer cell overexpression of podoplanin has been reported to promote lymphangiogenesis in a number of human carcinomas and mouse models of carcinogenesis [9,11,14,17]. We next investigated whether the deletion of cancer cell expressed podoplanin also affected the lymphatic vasculature in the two-stage chemical carcinogenesis model. First, we analyzed the normal vasculature by immunofluorescence stainings of ear sections stained for the blood vascular marker MECA-32 and the lymphatic vessel marker LYVE1. There were no major differences in the vascular morphology (Figure 3A1). The tissue area covered by blood vessels, stained for MECA-32, and lymphatic vessels, stained for LYVE1, showed no significant difference between the control and K5-Cre;Pdpn^flox/flox^ mice (Figure 3B). Next, we studied the lymphatic vasculature in the peritumoral area up to 500 µm from the tumor edge. Immunofluorescence analysis of tumors stained for LYVE1 and the pan-vascular marker CD31 (Figure 3A2) revealed a significant decrease of the LYVE1^+^ lymphatic area in the K5-Cre;Pdpn^flox/flox^ mice as compared to the control mice (Figure 3C). The decrease in the LYVE1^+^ area was associated with a reduced number of lymphatic vessels (Figure 3D). There was no significant change of lymphatic vessel size. We next investigated the potential contribution of tumor cell expressed podoplanin to the immune cell accumulation in tumors. Tumors were scored as either having strong, medium, low or no immune infiltration, using H&E-stained tissue sections. No significant difference between tumors in the control and K5-Cre;Pdpn^flox/flox^ mice was observed (Figure 3E). Quantification of wet lymph node weights did not show any significant difference between the control and K5-Cre;Pdpn^flox/flox^ mice (Figure 3F).

## 4. Discussion and Conclusions

Podoplanin expression is upregulated in a number of different human carcinomas, including SCCs of the skin, esophagus and head and neck, and has been reported as a biomarker for poor prognosis [3,6,9]. Additionally, expression of podoplanin by tumor cells is a marker of invasion in a number of human carcinomas [9,32,33]. While not normally expressed by the interfollicular epidermis, podoplanin has been reported to be upregulated early in the skin carcinogenesis process [17]. Moreover, tumor cell expressed podoplanin has been reported to act as an oncogene, promoting the tumorigenic and metastatic phenotype in epidermal keratinocytes [34], and silencing of podoplanin in podoplanin-expressing human oral SCC lines reduced the tumor formation capacity of xenografts in nude mice [35]. Together, these reports suggested an important role of keratinocyte-expressed podoplanin in promoting carcinogenesis. However, our findings in an epidermis-specific podoplanin knockout mouse model, using an established orthotopic multistep skin carcinogenesis model, revealed no differences in tumor initiation, multiplicity or malignant transformation. Thus, while increased expression of podoplanin might further promote carcinogenesis in some models, it is dispensable for multistep chemically-induced skin carcinogenesis. As a limitation of the present study, one has to keep in mind that the chemical carcinogenesis protocol used (DMBA for the induction of genetic alterations, TPA for tumor promotion), even though it has been previously widely used and is generally considered as one of the best characterized experimental mouse cancer models, does not fully reflect the pathogenesis of most cases of cutaneous SCC in humans. While chemical skin carcinogenesis was prominent centuries ago, ultraviolet B light exposure is considered to be the major pathogenetic factor for cutaneous SCC development today. Thus, it would be of interest to investigate the role of epidermis-expressed podoplanin in additional experimental SCC models in future investigations. Beyond the cancer aspect, our study confirms, in agreement with a recent report [5], that podoplanin expression is dispensable for the normal development and differentiation of the murine interfollicular epidermis, since we did not detect any major changes of the normal epidermal architecture in the K5-Cre;Pdpn^flox/flox^ mice.

Podoplanin is upregulated at the invasive front of a number of human cancers and mouse models of carcinogenesis, and has been reported to render cancer cells more motile, thereby facilitating the local invasion of host tissue [3,9,13,16]. Tumor cell expressed podoplanin at the invasive front of SCCs often co-localizes with the cell–cell adhesion molecule E-cadherin, promoting collective cell invasion [20,36]. However, in some areas of oral SCCs, podoplanin expression co-localized with reduced or virtually absent E-cadherin expression, promoting EMT and single cell invasion [12]. Similarly, podoplanin overexpression induced EMT in Madin-Darby canine kidney type-II epithelial cells and in immortalized HaCaT keratinocytes through the interaction with ERM proteins and upregulation of RhoA activity [19]. This is in agreement with our findings that single cell invasion was exclusively observed in Pdpn^flox/flox^ control mice, whereas collective cell invasion was detected in both the control and K5-Cre;Pdpn^flox/flox^ mice. This is also in line with our findings that primary keratinocytes of K5-Cre;Pdpn^flox/flox^ mice migrated faster compared to the controls, possibly due to reduced matrix adhesion [5]. The detailed mechanisms by which podoplanin regulates cancer cell migration are likely cell- and cancer type-specific, and their identification will require specific genetic deletions to better understand the contribution of different cell types in the microenvironment.

The metastasis of many human cancers, including SCCs of the head and neck, occurs predominantly through the lymphatic system, and the extent of lymph node involvement often determines the patient’s prognosis [37]. The overexpression of podoplanin has been found to promote peritumoral lymphangiogenesis in a number of human cancers and experimental animal models [9,11,34,38]. In agreement with these observations, we found reduced peritumoral lymphangiogenesis in the K5-Cre;Pdpn^flox/flox^ mice, even though there were no major changes in immune cell infiltration or draining lymph node weight. While the detailed factors responsible for the induction of lymphangiogenesis in the skin carcinogenesis model need to be investigated, a previous study using podoplanin-overexpressing tumor xenografts identified endothelin-1 to be upregulated in these tumors and also found that endothelin-1 promoted lymphatic endothelial cell proliferation in vitro [9]. Endothelin-1 was also suggested to contribute to cancer cell motility and invasiveness through the induction of tumor proteinases [39,40,41], highlighting a potential mechanism for how podoplanin-expressing cancers might acquire more aggressive phenotypes.

In conclusion, our results reveal for the first time that keratinocyte-expressed podoplanin is dispensable for the early skin carcinogenesis process. Since podoplanin is also upregulated by a number of non-epithelial stromal cells during early carcinogenesis, notably by dermal fibroblasts, these cells might compensate for the specific loss of podoplanin in keratinocytes. Since global podoplanin knockout mice are not viable, future studies with fibroblast-specific deletion of podoplanin might determine the specific contribution of fibroblast-expressed podoplanin in early carcinogenesis.
